# The combined effects of irradiation and herpes simplex virus type 1 infection on an immortal gingival cell line

**DOI:** 10.1186/1743-422X-11-125

**Published:** 2014-07-08

**Authors:** Aaro Turunen, Veijo Hukkanen, Michaela Nygårdas, Jarmo Kulmala, Stina Syrjänen

**Affiliations:** 1 Institute of Dentistry, Department of Oral Pathology, University of Turku, Lemminkäisenkatu 2, 20520 Turku, Finland; 2 Department of Virology, University of Turku, Kiinanmyllynkatu 13, 20520 Turku, Finland; 3 Department of Radiotherapy, Turku University Hospital, Clinic of Oncology, Hämeentie 11, 20521 Turku, Finland

**Keywords:** HSV-1, Herpesviruses, Irradiation, Oral cancer, Apoptosis, Immortal gingival cells, Radiation treatment

## Abstract

**Background:**

Oral mucosa is frequently exposed to Herpes simplex virus type 1 (HSV-1) infection and irradiation due to dental radiography. During radiotherapy for oral cancer, the surrounding clinically normal tissues are also irradiated. This prompted us to study the effects of HSV-1 infection and irradiation on viability and apoptosis of oral epithelial cells.

**Methods:**

Immortal gingival keratinocyte (HMK) cells were infected with HSV-1 at a low multiplicity of infection (MOI) and irradiated with 2 Gy 24 hours post infection. The cells were then harvested at 24, 72 and 144 hours post irradiation for viability assays and qRT-PCR analyses for the apoptosis-related genes caspases 3, 8, and 9, bcl-2, NFκB1, and viral gene VP16. Mann–Whitney *U*-test was used for statistical calculations.

**Results:**

Irradiation improved the cell viability at 144 hours post irradiation (P = 0.05), which was further improved by HSV-1 infection at MOI of 0.00001 (P = 0.05). Simultaneously, the combined effects of infection at MOI of 0.0001 and irradiation resulted in upregulation in NFκB1 (P = 0.05). The combined effects of irradiation and HSV infection also significantly downregulated the expression of caspases 3, 8, and 9 at 144 hours (P = 0.05) whereas caspase 3 and 8 significantly upregulated in non-irradiated, HSV-infected cells as compared to uninfected controls (P = 0.05). Infection with 0.0001 MOI downregulated bcl-2 in non-irradiated cells but was upregulated by 27% after irradiation when compared to non-irradiated infected cells (P = 0.05). Irradiation had no effect on HSV-1 shedding or HSV gene expression at 144 hours.

**Conclusions:**

HSV-1 infection may improve the viability of immortal cells after irradiation. The effect might be related to inhibition of apoptosis.

## Background

Herpes simplex viruses (HSV) are among the most common viral pathogens of the oral mucosa. Most of the oral HSV infections are caused by HSV-1 [1]. Symptomatic HSV reactivation causes cold sores affecting 15% of general population. Approximately 70% of the population sheds HSV-1 asymptomatically at least once a month, and many individuals appear to shed HSV-1 more than 6 times per month [2]. Reactivation of HSV-1 can be triggered by several factors such as stress, hormonal changes, dental treatment and other infections [1]. The oral mucosa is also frequently exposed to irradiation, because dental radiography comprises up to one-third of all the radiographic examinations undertaken in Nordic countries.

Reactivation of HSV-1 infection is frequent in oral cancer patients especially after chemoradiotherapy [3,4]. Globally, oral and lip cancer is the 15^th^ most common malignant tumor, with estimated 300.373 incident cases and 145.328 deaths annually [5]. Recently, HNSCC patients suffering from concomitant HSV-1 and human papillomavirus infections had the lowest survival rates after radiotherapy, less than one year since the primary diagnosis [6]. Due to whole-mouth exposure to carcinogens such as tobacco, premalignant cells are likely present in the clinically normal mucosa surrounding oral cancers at time of radiation treatment. Although HSV-1 has not been implicated in direct carcinogenesis of the oral cavity, HSV-1 infection and irradiation combined might be clinically relevant in the pathogenesis or recurrence of the head and neck cancers (HNSCC). These hypotheses prompted us to outline a concept that oral immortal keratinocytes infected with HSV-1 might be more resistant to apoptosis than the uninfected cells when irradiated.

Previous studies have shown that HSV infection can trigger and also block apoptosis in infected cells [7,8]. The extent of apoptosis after HSV-1 infection is thought to be cell type-related. It is likely regulated by different cellular factors such as caspases, bcl-2 family members and nuclear factor κB [7,9-14]. Caspases 3 and 9 have been reported to mediate HSV-1-induced apoptosis in human epithelial HEp-2 cells [15,16], whereas caspase 8 failed to do so [15], suggesting that HSV-1 induces apoptosis through the intrinsic, mitochondrial pathway [14]. Apoptosis is induced at very early stage of HSV infection [17,18], meanwhile the anti-apoptotic HSV genes represent genetic classes expressed during different phases of infection. Many of these anti-apoptotic factors of HSV-1 are encoded by late (γ) genes, including the protein kinase Us3 and glycoproteins gD and gJ, but also immediate-early (α) proteins such as ICP4 or ICP27 are important for blocking apoptosis [8,19-21].

The gene for the latency-associated (LAT) RNA has also anti-apoptotic activity [22]. We have previously shown that HSV-1 can cause a non-productive infection in epithelial cells where LAT RNA is expressed [23]. The present study was designed to assess the viability of immortal oral keratinocytes and the expression of apoptosis-related genes caspases 3, 8, and 9, bcl-2 and NF*κ*B1 during 144 hours after the HSV-1 infection with or without irradiation.

## Results

### Cell viability

**Figure 1:**
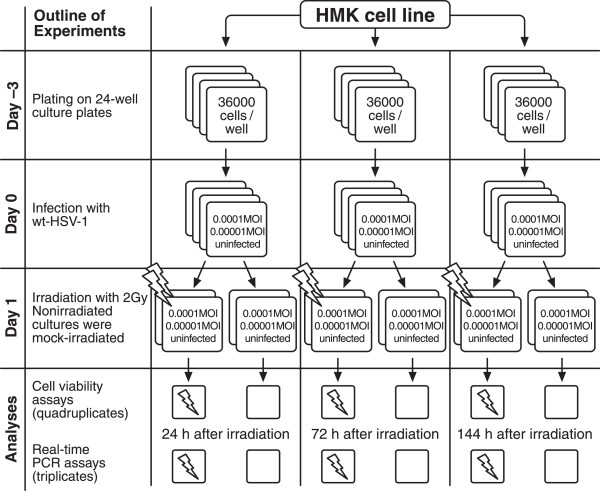
**The layout of a single experiment as described in the methods.** This experiment was repeated twice and also performed on HaCaT cells for the viability analyses.

**Figure 2:**
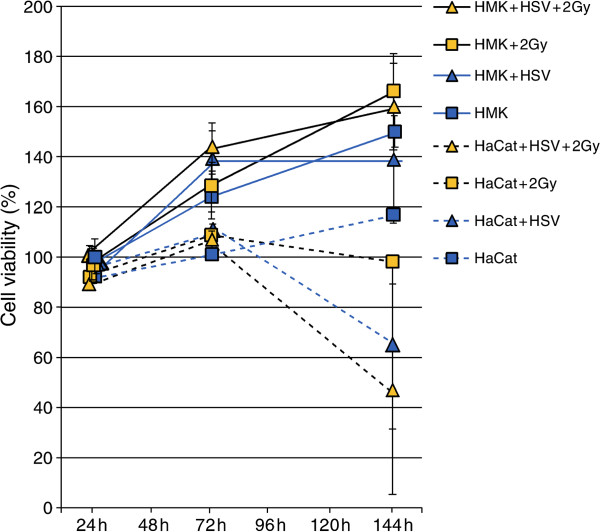
**The cell viability ratings for identically cultured HMK (straight line) or HaCaT (dashed line) cells.** The curves display data from HMK cell cultures obtained from a minimum of quadruplicate experimental cultures from two independent experiments and identical quadruplicate HaCaT cultures. Data represent uninfected cultures, cultures infected with 0.0001 MOI of HSV-1, cultures irradiated with 2 Gy of X-ray irradiation and cultures with combined 0.0001 MOI HSV-1 infection and subsequent 2 Gy X-ray irradiation treatment, measured at time points indicated post irradiation.

**Figure 3:**
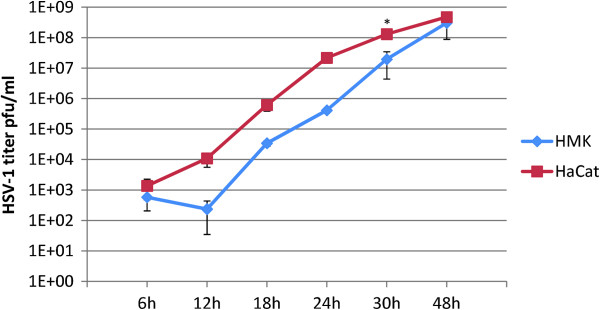
**HSV-1 shedding from HaCat- and HMK cell lines after HSV-1 infection at 5 MOI.** HSV-1 shedding into medium is measured using plaque titration assays on Vero cells. The data is derived from quadruplicate cultures measured at indicated hours post infection. Error bars represent SEM. * = p < 0.05 using the Mann–Whitney *U*-test.

**Figure 4:**
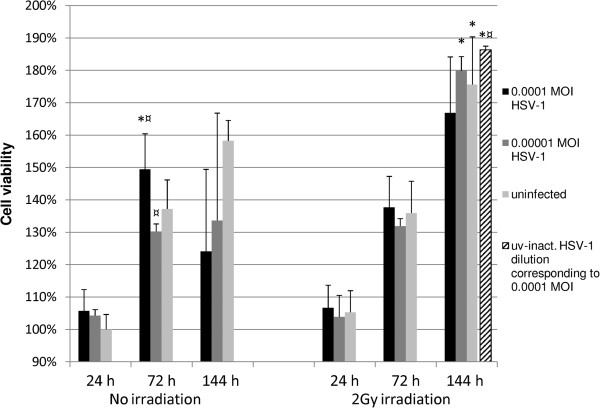
**Effect of HSV-1 infection and irradiation on HMK cell viability.** Viability ratings were measured from quadruplicate monolayer cultures from two independent experiments in 24-well plates and tested using Mann–Whitney *U*-test. Mean values are shown + SEM. The values are referenced against the 24 h, nonirradiated uninfected cultures which have a viability rating of 100%. The dashed bar shows the result of six cultures infected with UV inactivated HSV-1 at 0.0001 MOI. This percentage result is from a separate experiment and is compared with its own appropriate uninfected, nonirradiated controls (p = 0.01). * = p ≤ 0.05 when comparing irradiated and non-irradiated cultures and ¤ = p ≤ 0.05 when uninfected and infected cultures are compared from the same time point.

To study the long-term effects of HSV-1 infection, we used low MOI infections to avoid excessive cell death due to overwhelming HSV-1 infection at the end of the study i.e. at 144 hours. First, the effects of HSV-1 infection and irradiation on the viability ratings of oral immortal keratinocytes (HMK cells) were analyzed. The outline of the experiments is shown in Figure 1. HaCaT cells (spontaneously immortalized skin keratinocytes) were used as controls in these experiments, because HSV-1 is known to reduce their viability. Figure 2 summarizes the results of HMK and HaCaT cell viability assays after HSV-1 infection and irradiation. As expected, both the irradiation with 2 Gy and HSV-1 infection with 0.0001 MOI reduced the HaCaT cell viability at 144 hours (P = 0.021). The effect was synergistic in that HaCaT cultures infected with HSV-1 and irradiated were the least viable. In contrast, the viability of HMK cells improved after HSV-1 infection combined with irradiation, and irradiated HMK cells had the highest viability among all experiments (P = 0.05).To ensure that our findings were not caused by differences in viral permission between the two cell lines we performed a separate 48-hour experiment comparing the viral replication kinetics between the HMK and HaCat cell lines infected with 5 MOI. No statistically significant differences in viral infectious titers were present at the end of the 48-hour culture (P = 0.57, Figure 3).Figure 4 summarizes the HMK cell viability data according to the HSV-1 viral load at infection. At 24 hours post irradiation, no differences in the viability of HMK cells were found irrespective of HSV status or irradiation. At 72 hours, non-irradiated cultures infected with 0.0001 MOI of HSV1 were statistically significantly more viable than non-irradiated uninfected control cells or irradiated cells infected with the same MOI (p = 0.05). However, the viability dropped at 144 hours, when also the cytopathic cellular changes caused by HSV infection were prevalent.

Interestingly, the viability of the irradiated HMK cells at 144 hours was significantly higher compared to that of their non-irradiated counterparts (p = 0.05). The viability of the irradiated cells was approximately 11% and 34% higher in the uninfected and infected (0.00001 MOI) cultures, respectively.

**Figure 5:**
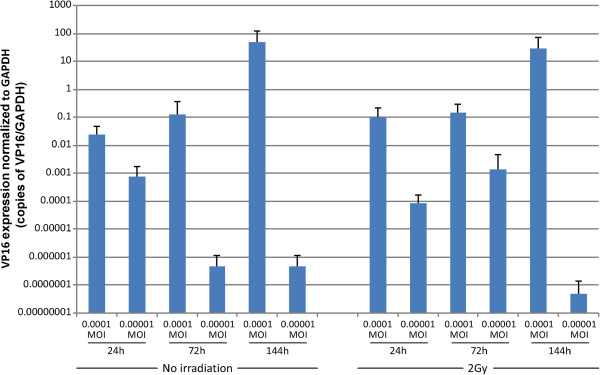
**HSV-1 VP16 expression.** VP16 expression is shown in a logarithmic scale and measured in triplicates from triplicate monolayer cultures. These assays are done from the same samples from which also the TaqMan® -gene expression assays were performed. The log scale shows the normalized VP16 copy number/GAPDH copy number. Mean values are shown + SEM. Testing was done using the Mann–Whitney *U*-test comparing the irradiated and non-irradiated groups. No statistically significant differences were found between samples. General linear modeling found no significant effect of 2 Gy irradiation on HSV-1 VP16 expression (p = 0.614, R squared = 0.046).

**Figure 6:**
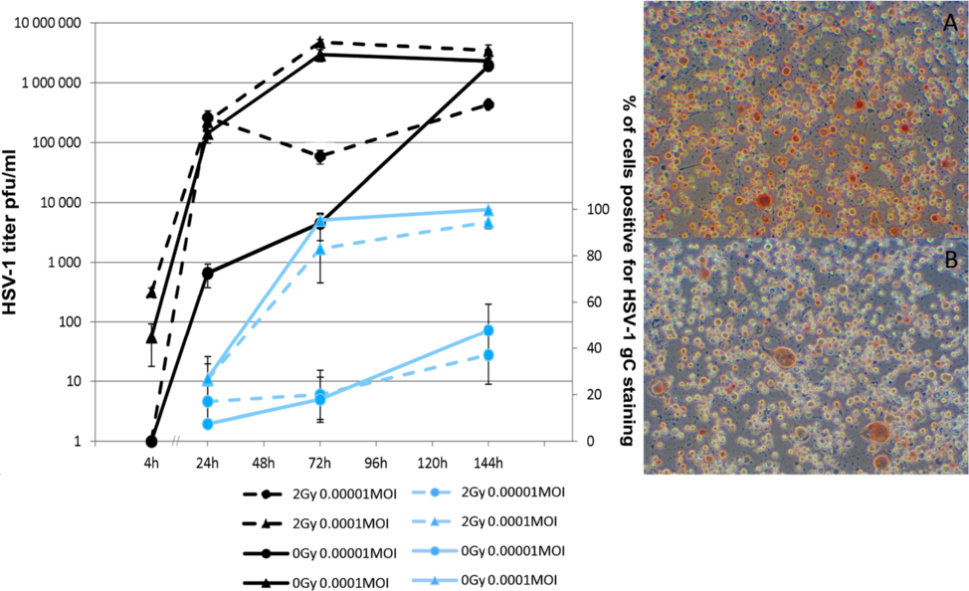
**The results of HSV-1 gC immunoperoxidase (IPS) staining (blue bars) and HSV-1 titer (black bars).** The graph shows HMK cells, infected with HSV-1 at MOI shown and irradiated 24 hours post infection. The measurements were done from medium samples taken from the cultures at times of fixation for IPS at designated hours post irradiation. HSV-1 titer was measured using standard plaque titration assays on b-Vero cells. The numbers represent the averages of a minimum of quadruplicate cultures +/- SEM. The values for IPS staining were averaged from the results of two independent observers. The photomicrographs, taken at 400 × from a random location on the culture, present HMK cells infected with 0.0001MOI HSV-1 and irradiated **(A)** or nonirradiated **(B)** and fixed for IPS at 144 hours post irradiation. No significant differences in IPS staining or virus production were found when irradiated- and nonirradiated cultures infected with the same MOI were compared (Mann–Whitney *U*-test, p value range from p = 1 to p = 0.121).

At 144 hours, the irradiated cultures infected with UV inactivated HSV-1 at 0.0001 MOI displayed a 17% elevation in their viability rating when compared to that of the uninfected cultures and the irradiated cultures infected with 0.00001 MOI (p = 0.01).The HSV VP16 expression increased from 24 hours to 144 hours, indicating the progression of HSV-1 infection (Figure 5). Irradiation had no effect on VP16 expression. No correlation between irradiation and HSV-1 VP16 expression was found by univariate general linear modeling (P = 0.61, R squared = 0.046).The kinetics of low-MOI HSV-1 infection with 0.0001 MOI and 0.00001 MOI in HMK cells was also studied by using immunoperoxidase staining (IPS) for HSV-1 gC and standard HSV-1 plaque titration assays from medium samples. At 144 hours, the cultures infected with 0.0001 MOI or 0.00001 MOI were, on average 97% or 42% infected, respectively. The highest titers of HSV-1 were observed in irradiated 0.0001 MOI cultures at 144 hours. There was no statistically significant difference in HSV-1 gC expression or virus production between the irradiated- and nonirradiated cultures infected with the same MOI (p value range from p = 1 to p = 0.121, Figure 6).

### Apoptosis-related gene expression

#### NFkappaB1

**Figure 7:**
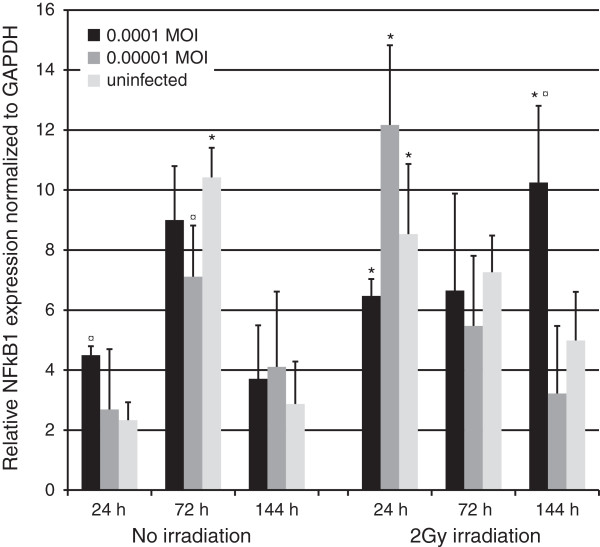
**NFκB1 expression.** NFκB1 expression was measured in triplicates from triplicate monolayer cultures by qRT-PCR at 24, 72 and 144 hours post irradiation with 2 Gy and tested using Mann–Whitney *U*-test. Mean values are shown + SEM. The expression was calculated relative to GAPDH mRNA levels (NFkappaB/GAPDH). * = p ≤ 0.05 when comparing irradiated and non-irradiated cultures and ¤ = p ≤ 0.05 when uninfected and infected cultures are compared from the same time point.

HSV infection at the highest MOI (Figure 7, p = 0.05) or irradiation with 2 Gy increased NF*κ*B1 expression of HMK cells at 24 hours (p = 0.05 for both). NFκB1 expression increased further by combined effects of HSV-1 infection and irradiation (p = 0.05). At 72 hours, non-irradiated HSV-1 infected cells displayed a slight downregulation of NF*κ*B1 expression not observed in the irradiated HSV infected cultures. At 144 hours, the combined effects of HSV infection (highest MOI of 0.0001) and irradiation led to a significant upregulation in NF*κ*B1 expression (p = 0.05).

#### Bcl-2

**Figure 8:**
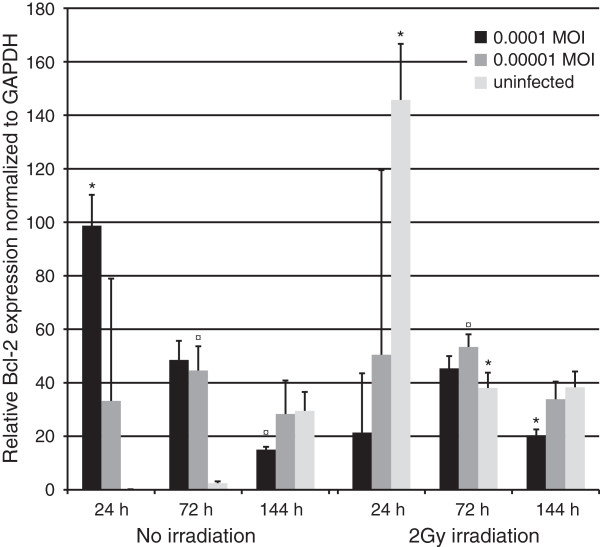
**Bcl-2 expression.** Bcl-2 expression was measured in triplicates from triplicate monolayer cultures by qRT-PCR at 24, 72 and 144 hours post irradiation with 2 Gy and tested using Mann–Whitney *U*-test. Mean values are shown + SEM. The expression was calculated relative to GAPDH mRNA levels (bcl-2/GAPDH). * = p ≤ 0.05 when comparing irradiated and non-irradiated cultures and ¤ = p ≤ 0.05 when uninfected and infected cultures are compared from the same time point.

Bcl-2 expression increased nearly 1000-fold after infection with HSV-1 or irradiation (Figure 8, p = 0.05) at 24 hours, but the combined effects seemed to compensate this upregulation. At 72 hours, upregulation of bcl-2 expression was still present in the irradiated cultures but disappeared at 144 hours. At 144 hours, HSV-1 infection with 0.0001 MOI downregulated bcl-2 expression in non-irradiated cells but irradiation upregulated it by 27% compared to nonirradiated cultures infected with the same MOI (P = 0.05).

#### Caspase 8

**Figure 9:**
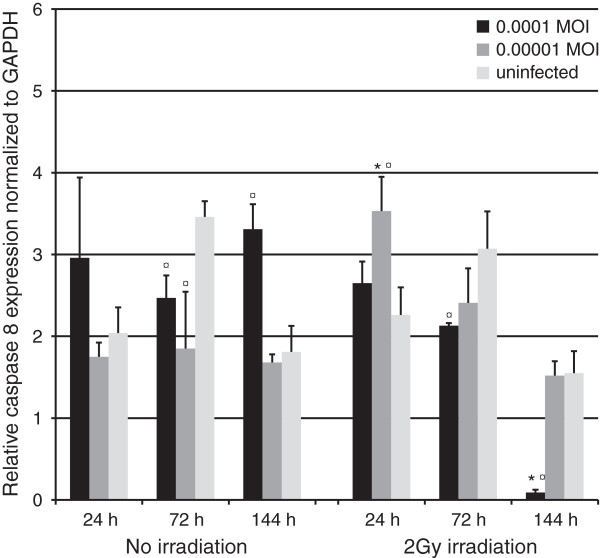
**Caspase 8 expression.** Caspase 8 expression, as measured in triplicates from triplicate monolayer cultures by qRT-PCR at 24, 72 and 144 hours post irradiation with 2 Gy and tested using Mann–Whitney *U*-test. Mean values are shown + SEM. The expression was calculated relative to GAPDH mRNA levels (Caspase 8/GAPDH). * = p ≤ 0.05 when comparing irradiated and non-irradiated cultures and ¤ = p ≤ 0.05 when uninfected and infected cultures are compared from the same time point.

Caspase 8 expression was not statistically significantly altered at 24 hours except in the irradiated cultures infected with 0.00001 MOI where an upregulation was observed (Figure 9, p = 0.05). At 72 hours, cultures infected with the highest MOI displayed a lower expression of caspase 8 (p = 0.05) in both the irradiated and non-irradiated cultures, when compared to their respective controls. Also, downregulation was observed in non-irradiated cultures infected with the lowest MOI (p = 0.05). However, at 144 hours, non-irradiated cultures infected with the highest MOI had an increase in caspase 8 expression (1.83 -fold upregulation, p = 0.05), whereas a significant downregulation (20.9-fold) was observed in their irradiated, HSV infected (highest MOI) counterparts (p = 0.05).

#### Caspase 9

**Figure 10:**
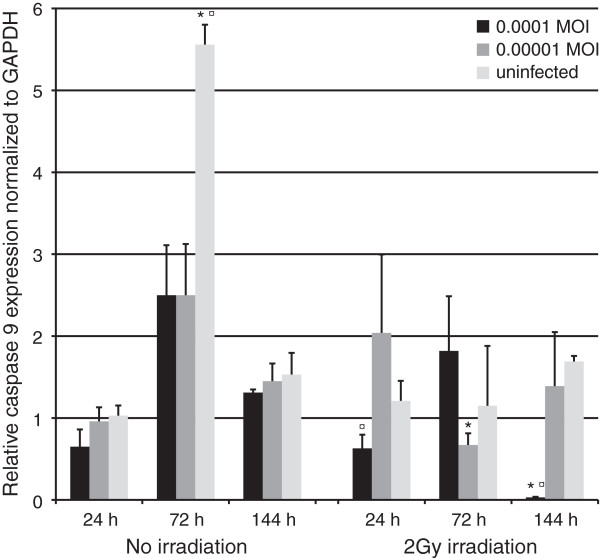
**Caspase 9 expression.** Caspase 9 expression, as measured in triplicates from triplicate monolayer cultures by qRT-PCR at 24, 72 and 144 hours post irradiation with 2 Gy and tested using Mann–Whitney *U*-test. Mean values are shown + SEM. The expression was calculated relative to GAPDH mRNA levels (Caspase 9/GAPDH). * = p ≤ 0.05 when comparing irradiated and non-irradiated cultures and ¤ = p ≤ 0.05 when uninfected and infected cultures are compared from the same time point.

At 24 hours, caspase 9 expression was downregulated in HMK cells irradiated and infected with HSV-1 at 0.0001 MOI (Figure 10, p = 0.05). At 72 hours, an upregulation in caspase 9 expression was observed in non-irradiated control cells and the expression was lowest in the irradiated cultures infected with the lowest MOI (p = 0.05). At 144 hours, the cultures irradiated and infected with the highest MOI downregulated caspase 9 expression by 53.5-fold (p = 0.05).

#### Caspase 3

**Figure 11:**
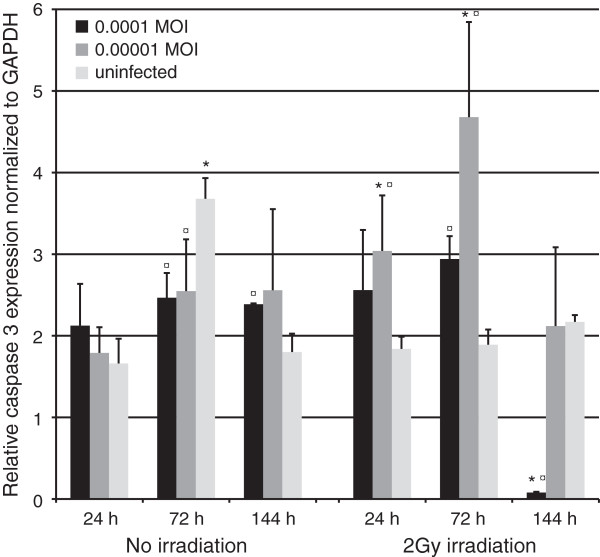
**Caspase 3 expression.** Caspase 3 expression, as measured in triplicates from triplicate monolayer cultures by qRT-PCR at 24, 72 and 144 hours post irradiation with 2 Gy and tested using Mann–Whitney *U*-test. Mean values are shown + SEM. The expression was calculated relative to GAPDH mRNA levels (Caspase 3/GAPDH). * = p ≤ 0.05 when comparing irradiated and non-irradiated cultures and ¤ = p ≤ 0.05 when uninfected and infected cultures are compared from the same time point.

At 24 hours, caspase 3 expression was upregulated by HSV infection but significantly only in irradiated cultures infected with the lowest MOI (Figure 11, P = 0.05). Also, the expression was higher in irradiated and infected cells than in their non-irradiated counterparts (P = 0.05). At 72 hours, the irradiated cells showed lower caspase 3 expression and HSV-1 infection downregulated caspase 3 in nonirradiated cultures (p = 0.05 for both). However, caspase 3 expression increased in HSV infected cells after irradiation (p = 0.05). At 144 hours, HSV infection with the highest MOI resulted in a significant increase in caspase 3 expression (1.3 –fold upregulation, P = 0.05) while irradiation of these infected cells downregulated caspase 3 expression by 23-fold at the same time point (p = 0.05).

#### ICP27

**Figure 12:**
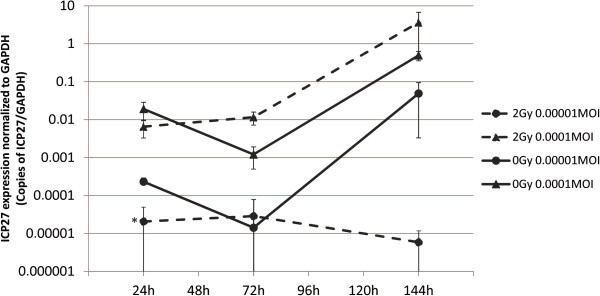
**HSV-1 ICP27 expression.** The expression of HSV-1 immediate early gene ICP27 as measured by qRT-PCR at 24, 72 and 144 hours post irradiation with 2 Gy. Normalized values are copies of ICP27/copies of GAPDH and are shown on a log scale. Error bars are +/-SEM. Statistical analysis was performed using a Mann–Whitney *U*-test (* = p < 0.05).

ICP27 expression increased from 24- to 144 hours except for irradiated 0.00001 MOI cultures where it decreased slowly. At 24 hours, the irradiated cultures infected with 0.00001 MOI had lower ICP27 expression than their nonirradiated counterparts (p = 0.03). Irradiated cultures infected with 0.0001MOI displayed the highest amount of ICP27 at 144 hours (Figure 12).

## Discussion

This study was conducted to characterize the combined effects of irradiation and HSV-1 infection on an immortal oral epithelial (HMK) cell line. The most important result was that after 144 hours in culture, irradiation lead to increase in the viability of this immortal cell line and the effect was potentiated by HSV-1 infection in cells infected with a low MOI. Simultaneously, the expression of caspases 3, 8, and 9 was downregulated in HSV-1 infected and irradiated cells, but bcl-2 was upregulated. This cannot be due to the overall general shutoff of gene expression caused by progressive HSV-1 infection, as nuclear factor *κ*B was significantly upregulated in this time point compared to the nonirradiated infected cells. This primary observation suggests that HSV-1 infection and irradiation, to both of which oral epithelial cells are frequently exposed, might aid transformed cells to resist the toxic effects of HSV-1 infection and even gain a viability advantage. Downregulation of caspases essential in apoptosis might be one of the pathways involved in this effect. Although this is still too short a time to establish conclusions with regard to *in vivo* tumorigenesis, these *in vitro* results warrant further studies because they can be clinically important. The gingival tissue around the teeth is frequently exposed to ionizing radiation during dental treatments and the effect can be even potentiated by the scattered irradiation due to metallic implants or fillings [24]. Also, reactivation of HSV-1 infection and viral shedding in saliva is frequent. Even more important is that the presence of HSV-1 in HNSCCs might affect patient survival after treatment with surgery with radiotherapy or chemoradiotherapy [6].

The assay used to study the cell viability is based on ATP detection that correlates with the number of living cells [25,26]. HSV can enhance the glycolysis of the infected cell but this effect is not a significant source of upward bias in these experiments, as demonstrated by data from Peri et al. where uninfected cells were compared to several HSV-1 mutant- and wild-type strains [14]. Also, ATP is quickly degraded outside the cell and therefore already dead cells cannot increase the “viability” ratings of the culture analyzed.

### Immortal cell lines

In the present study HSV-1 infection and irradiation had near-opposite effects on cell viability of HaCaT cells and HMK cells. This can be partly explained by the origin of these two cell lines, skin (HaCaT) and oral mucosa (HMK), and/or the differences in their genome. Although both are spontaneously immortalized nontumorigenic keratinocyte cell lines, HMK cells could be considered more abnormal than HaCaT cells, presenting a totally tetraploid karyotype (DNA index 2.01). Spontanously immortalized HaCaT cells were found to be hypotetraploid with a DNA index of 1.92 [27,28]. It is impossible to obtain karyotypically similar two cell lines However, there are no earlier comparative studies on radioresistance, permissivity to virus infections or cell viability done with different spontaneously immortal cell lines. Although genetically quite different, these two cell lines support similar rates of HSV-1 infection as shown here although the replication starts slower in the HMK cells.

One of the limitations of this study is that the cells were around 80% confluent at the time of infection (or mock infection) and continued to increase in number until past the 24 hours post-irradiation time point. Caspase and NFκB1 expression was found to be increased in the uninfected and nonirradiated HMK cells at 72 hours. There is a possibility that supraconfluency of the cultures was reached somewhere between the 24 hours and 72 hours, which might induce differentiation-related signals to which caspases also belong in human keratinocytes [29]. Apparently, these effects have dissipated by the 144 hour time point since the caspase levels in uninfected and nonirradiated cells return to the 24-hour levels. Also, this effect was not seen in irradiated cells but as irradiation has profound effects on differentiation-related signaling, the effect of confluency on 72 hour nonirradiated cultures remains likely [30].

### The effect of irradiation

Transcriptional activation of cell death regulatory genes is of the utmost importance for cellular radiosensitivity [31]. BcI-2 has been shown to protect cells from irradiation-induced cell death [32]. On the contrary, NF*κ*B1 is activated by the ATM kinase following irradiation [33] and confers resistance to apoptosis which can be abrogated by blocking NF*κ*B1, leading to cytotoxicity and caspase 3 activation in cancer cell lines after irradiation [34]. Irradiation affected the uninfected HMK cells by inducing NFκB1 and Bcl-2 expression at 24 hours but no effect was seen on caspase expressions. The Bcl-2 upregulation continued up to 72 hours when NFκB1 and caspase 9 and 3 expressions declined. The effects of irradiation on uninfected cells’ gene expression mainly dissipated by 144 hours. By this time, however, increased cell viability was found in irradiated cells, which reflect an increased resistance to radiation-induced damage. This could be explained by earlier upregulation of the antiapoptotic genes and lower activity of the mitochondrial apoptotic pathway indicated by the lower expression levels of caspases 9 and 3 at 72 hours and increase in the Bcl-2 expression from 24 to 72 hours. We found also that NFκB1 was upregulated 24 hours postirradiation but returned back to baseline level at 144 hours. It might be that upregulation of NFκB1 found at 24 hours is due to the genotoxic stress which allows DNA damage repair and cell survival as shown by Janssens et al., [35].

### The effects of HSV-1

A lytic HSV-1 infection almost always destroys its host cell. However, there is evidence that HSV-1 may latently or nonproductively infect the epithelial cells as well [23]. Accordingly, HSV-1 infection together with other cofactors like irradiation might cause changes in spontaneously immortal cells toward malignancy. HSV-1 infection has been found to activate the NF*κ*B1 transcription factor to prevent the target cell from undergoing apoptosis [36]. Interestingly, our results pointed out that HSV-1 elevated Bcl-2 and NFκB1 expression already at 24 hours after mock-irradiation but there were not yet any effects on cell viability. As the infection progressed, caspases 3, 8 and 9 were downregulated compared to the uninfected cultures at 72 hours, surprisingly together with NFκB1, but the Bcl-2 levels were increased. This may be due to the accelerating spread of HSV-1 in the cultures at this time point, leading first into a well-known evasion of apoptosis caused by the expression of typical HSV-1 antiapoptotic proteins gD, gJ, Us3, ICP27 and ICP4 until the infection nears its completion at 144 hours [8,19-21]. The lowest MOI causes more robust changes in NFκB1 and Bcl-2 levels as demonstrated by the lack of statistical significance in the changes in expression levels found with higher-MOI, although the higher-MOI expression levels displayed a trend in the same direction. Aubert et al. implied that HSV-1 blocks apoptosis by targeting Bax and thus preventing mitochondrial cytochrome *c* release and therefore activation of caspase 9 in human epithelial cells [15]. Bcl-2 can act as an inhibitor of Bax [31,37,38]. Therefore in HSV-1 infected cells, upregulated Bcl-2 might heterodimerize with Bax and block apoptosis, similarly as described earlier in non-irradiated cells [15].

Our results showed that HSV-1 infection at 144 hours had progressed to a point where most of the cells were nearly completely infected. HSV-1 infection is thought to trigger caspase 9-mediated apoptosis, caspase 3 being important for the downstream apoptotic pathway [13,16]. It has been suggested that HSV-1 induces apoptosis by first triggering the cytochrome c release from the mitochondria, thus activating caspase 9 leading to apoptosome formation and caspase 3 cleavage [15]. However, our results showed that caspases 8 and 3, but not caspase 9, were upregulated at 144 hours due to HSV-1. This difference may be caused by the differences in the cell lines studied, as our HMK cells are HPV negative, and the epithelial cells used by Aubert et al. are known to be HeLa contaminants containing HPV-18 which affects apoptosis, especially via caspase 8 [39,40].

### The combined effects of HSV-1 and irradiation

The most intriguing aspect of our data stems from the major differences in gene expression and viability responses of the cultured cells when the combined effects of irradiation and HSV-1 infection are compared to the effects of either exposure only. As seen in our results, at 144 hours the HSV-1 infection has spread to most of the cells in culture. Therefore the HSV-1 mediated antiapoptotic effects are best represented in the 144 hour results as the previous time points are less representative of the HSV-related effects. The combined effects of HSV-1 and irradiation did not cause any additional toxicity as determined by the viability assays. Irradiation of HSV-1 infected cells resulted in upregulation of caspase 3, caspase 8 and downregulation of caspase 9 at 24 hours. Simultaneously, NFκB1 was upregulated in all irradiated cultures compared to their nonirradiated counterparts regardless of HSV-1 presence. Therefore the immediate NFκB1 response to radiation does not seem to be affected by HSV-1. At 72 hours, bcl-2 and caspase 3 were upregulated and caspases 8 and 9 downregulated. Interestingly all caspases were downregulated at 144 hours while both NFκB1 and bcl-2 were upregulated. Since ICP27 is important in prevention of apoptosis [8], it is tempting to speculate whether ICP27 plays a role in the effects found here, partly because the highest ICP27 expression was detected at 144 hours in the irradiated infected cultures. Therefore, the role of ICP27 in irradiation induced apoptosis needs further study. In our experiment, HSV-1 infection was largely unaffected by irradiation as determined by VP16 qRT-PCR, virus culture and staining for HSV-1 gC. This would indicate that at 2 Gy, HSV-1 survives the irradiation and its infection rate remains unaffected.

Recently Dufour et al. [41] showed that HSV-1 ribonucleotide reductase R1 (rR) protects cells from apoptosis by binding to caspase 8. Spear et al. [42] reported that infection with rR –defective HSV-1 leads to increased apoptosis as measured by FACS analysis. When their results at 72 hours post irradiation are examined more closely, the tumor cells infected with HSV-1 had double the amount of apoptosis than the cells infected with the same virus but combined with 2 Gy irradiation. 2 Gy irradiation in itself had a negligible effect on apoptosis in their experiment. In the present study, contrary to the results of Spear et al. at the same time point, apoptotic gene expression was not present at high levels and no effects in cell viability were observed before the 144 hour time point, not included in their data.

After 144 hours in culture, cell viability was gradually lowering in nonirradiated infected cultures and apoptotic markers caspase 3 and 8 upregulated together with a decline in bcl-2 due to advanced HSV-1 infection, supporting the current literature on HSV-1 related apoptosis [13-16,42]. However, the most striking effect observed in this study is that irradiation of HMK cells with 2 Gy with or without HSV-1 infection does not actually lower the viability of the cells or lead to outright cell death during the study period and even lead to an elevation in cell viability. The combined effects exerted a profound abrogation of the expression of all caspases studied while NFκB1, that until 144 hours had remained relatively constant, strongly upregulated implicating the NFκB1 pathway as a mediator of long-term radiation responses in HSV-1 infected cells. NF*κ*B1 has diverse roles in cellular apoptosis [43] and inhibition NF*κ*B has been linked to apoptosis and delayed cell growth [44]. Therefore its upregulation may have contributed the effects we observed. The NF*κ*B1 pathway, when activated, leads to higher bcl-2 expression and therefore lower expression of apoptotic markers such as caspase 3 [45]. This is clearly supported by our findings in irradiated and infected cells. Bcl-2 is implicated in resistance to radiation therapy and chemotherapeutic agents [32,46]. Its expression displayed a downward trend in time, but remained at a higher level by the end of the experiment in irradiated cells, particularly those infected with 0.0001 MOI. This implies that bcl-2 may contribute to the observed downregulation of the intrinsic apoptotic pathway.

### Innate immunity

Because effects on cell viability were also seen using a UV inactivated virus that causes no visible HSV-1 plaque formation, it is possible that these effects might be at least partly mediated by the effects of innate immunity. This would be plausible, given that the tissue wouldn’t need to be completely infected with HSV to have far reaching effects. However, the presence of HSV-1 would still be required. Irradiation induces a wide variety of innate immunity related genes such as TNF-α and IFN-γ [47]. TNF-α has been linked to radioresistance of oral cancer cells whereas IFN-γ is able to induce cathepsin S expression which leads to radioresistance [48,49]. These factors could contribute to the effects observed in our study.

#### Summary

To summarize, after six days in culture the combined effects of HSV-1 infection and 2 Gy irradiation lead to an increase in NF*κ*B1 and bcl-2 expression, significantly lower expression of caspases 3, 8, and 9 and higher viability ratings as compared to the non-irradiated infected cultures, but also seen using a UV-inactivated virus. Apoptotic pathways are possibly involved in these effects. As oral epithelial cells are coexposed to HSV-1 infection and radiation during radiotherapy or dental radiographic exposures, there might be an increased risk for cellular transformation in subjects who are exposed to other common carcinogens, such as tobacco and alcohol. Future studies are needed to explore the significance of the present results in clinical settings.

## Methods

### Cell culture

Spontaneously immortalized human gingival keratinocytes (HMK) used in the experiments (Figure 1) were kindly provided by Dr. M. Mäkelä, University of Helsinki, Finland [27]. The cells were thawed from liquid nitrogen and grown in 80 cm^2^ Nunclon flasks (Sigma-Aldrich, St. Louis, MO, USA) for four passages before being trypsinized and plated in 24-well plates (Nunc, Roskilde, Denmark). Cells from passage 27 were used for the experiments. The cells were grown in Keratinocyte Serum-free Medium (KSFM by Gibco, Grand Island, NY, USA) supplemented with human recombinant epidermal growth factor (0.1-0.2 ng/ml) and bovine pituitary extract (20-30 μg/ml). For viability assays, HaCaT cells [28] (obtained from CLS Cell Lines Service GmbH, Eppelheimer, Germany) of passage 16 were also used to compare their response to irradiation and 0.0001 MOI HSV-1 infection to that of HMK cells. HaCaT cells were cultured in Dulbecco’s modified Eagles medium (D-MEM) with 10% inactivated fetal bovine serum (FBS).

### HSV-1 infection

The cells were seeded to 24-well plates at 36000 cells/well. 70% confluence was achieved in two days (Figure 1). At this time point, the cells were infected with wild-type HSV-1 (strain 17+) at two different low viral loads simulating natural HSV-infection: 1) 0.0001 MOI and 2) 0.00001 MOI. Uninfected cells served as controls. The viral dilutions were verified by plaque titration on Vero cells. The experimental infections were performed by replacing the growth medium with 300 μl of D-MEM supplemented with 7% inactivated FBS for HaCat cells or standard KSFM for HMK cells, with HSV-1 at the required MOI. After one hour the infection medium was removed and replaced with 1 ml of KSFM for HMK- and DMEM for HaCat cells. Then, the medium was replaced every 3 days during the 6-day experiment.

### Infection with UV inactivated HSV-1

A subset of the same HSV-1 stock as described above was UV-inactivated using a standard protocol. Wild-type HSV-1 was UV-irradiated for 30 min on ice, resulting in a 10E3 fold reduction in titer. This viral stock was used and diluted as the wild-type HSV-1 for 0.0001 MOI infections.

### HSV-1 replication kinetics between HaCat and HMK cells

The two cell lines were cultured in 24-well plates for three days in their respective culture media (see above) until near-confluent monolayers were reached. The wells were then infected with wild-type HSV-1 (strain 17+) at 5 MOI using a similar protocol as described above. Thereafter, one plate with quadruplicate cultures for each cell line was harvested and medium samples were collected at 6-hour intervals until 30 hours’ time point. Additional plates were harvested at 48 h post infection. The shedding of HSV-1 into medium samples was subsequently determined using a standard quadruplicate plaque titration assays on Vero cells.

### Irradiation

One day after infection, the relevant experimental cultures were irradiated at Turku University Hospital (Department of Oncology and Radiotherapy) using a linear accelerator (Clinac 2100C/D, Varian Medical Systems, Palo Alto, CA) at a total dose of 2 Gy of 6 MV x-ray irradiation at a dose rate of 3 Gy/min. Mock-irradiated cell cultures were included in the experiment (Figure 1).

### Viability assays

The viability of the cells was determined with CellTiter-Glo® Luminescent Cell Viability Assay (Promega, Madison, WI, USA) at 24, 72 and 144 hours after irradiation [14]. In order to match the viability and gene expression experiments, exactly the same culturing conditions were used. The viability assays were performed in 24-well plates, using the following modified protocol as recommended by Promega technical support after consultation: One half of the medium volume (500 μl) was replaced by 500 μl of CellTiter-Glo reagent to achieve the recommended 50/50 medium/reagent rate. The plates were then shaken using an orbital shaker at a low speed for 2 minutes followed by incubation in dark at room temperature for 10 minutes. After the incubation, 200 μl from each experimental well was pipetted into 96-microplate wells (Culturplate 96 White, Perkin Elmer, MA, US) for analysis in a luminometer (Wallac Victor3 1420, Perkin Elmer) according to manufacturer’s instructions. The functionality of the assay in this setting was validated separately (data not shown). Every plate included triplicate medium samples and empty wells for negative controls, along with quadruplicate experimental assays for every MOI used and the uninfected control cells of that time point with or without irradiation.

### RNA extraction

At 24, 72 and 144 hours after irradiation, the cells were harvested into Trizol reagent (Invitrogen, Paisley, UK) and RNA was extracted according to manufacturer’s instructions.

### cDNA synthesis and real time RT-PCR

First-strand cDNA was synthesized using First-strand cDNA Synthesis Kit (Applied Biosystems, Foster City, CA, US) and total RNA as a template. The cDNA synthesis was performed according to the manufacturer’s instructions. Real-time RT-PCR (TaqMan) reactions were performed in a reaction volume of 20 μl containing 25 - 100 ng of cDNA with TaqMan Universal PCR MasterMix and TaqMan® Gene Expression assays (Applied Biosystems) for NF*κ*B1 (manufacturer’s identification number Hs00765730_m1), Bcl-2 (Hs00608023_m1) and Caspase 3 (Hs00154261_m1), Caspase 8 (Hs01018151_m1) and Caspase 9 (Hs00154261_m1) using GAPDH (Hs02758991_g1) for normalization. The reactions were performed in triplicate runs from triplicate analyses and repeated twice, using a 7900HT Fast Real-Time PCR System (Applied Biosystems, Foster City, USA). The reaction conditions were 2 min at 50°C, 10 min at 95°C, and a two-step cycle of 95°C for 15 s and 60°C for 60 s for a total of 40 cycles. Each run included a dilution series of 400 ng to 12.5 ng of cDNA from HMK and HaCat control samples for standard curves. In addition, three no-template control reaction mixtures were added in every run. The amplification curves and standard curves were drawn and analyzed using the manufacturer’s software SDS2.3 and Microsoft Excel 2010. The averages were calculated from every triplicate analysis and the results were normalized against the GAPDH housekeeping gene mRNA levels (Applied Biosystems), except for HSV gene expression (VP16 and ICP27), where in-house GAPDH was used. The quantitative VP16 (α-TIF) mRNA RT-PCR was done as described previously [50] using the primers for HSV-1 VP16 as described by Broberg et al. [51]. The quantitative ICP27 (UL54) mRNA RT-PCR was done similarly, using the primers for ICP27 (Paavilainen H et al. unpublished data).

### Immunoperoxidase staining

First, the cells were cultured in 24-well plates as described above. At 24-, 72- and 144 hours post irradiation, medium samples were first drawn from the culture plates for subsequent plaque titration assays, then the cells were washed in PBS, fixated in 4°C methanol, washed with PBS-Tween 20 and stained against HSV-1 glycoprotein C, using a protocol modified from Ziegler et al. [52,53]. The results were read by two independent observers and are presented as an average from these observations. The sample photomicrographs from these observations for Figure 6 were taken at 400 × magnification on a darkfield setting using Leica DC500 camera with Leica application suite v4.2 (Leica Microsystems GmbH, Wetzlar, Germany). No editing of the pictures was done.

### Statistical analysis

Statistical significances of the results were analyzed with the Mann–Whitney *U* test, using SPSS 19 with SPSS advanced statistical package (IBM SPSS Statistics for Windows, Version 19, Armonk, NY: IBM Corp. Released 2010). Univariate general linear modeling was used to determine whether irradiation had a general effect on HSV-1 VP16 expression. The p-values equal to, or lower than 0.05 were considered to be statistically significant.

## Abbreviations

HSV-1: Herpes Simplex Virus type 1; KSFM: Keratinocyte Serum-free medium; D-MEM: Dulbecco’s modified Eagle’s medium; MOI: Multiplicity of infection; GAPDH: Glyceraldehyde-3-phosphate dehydrogenase; PBS: Phosphate buffered saline.

## Competing interests

The authors declare that they have no competing interests.

## Authors’ contributions

AT contributed to the design of the studies, performed the major part of the experiments, performed the statistical analyses and prepared the manuscript. MN participated in data analysis and drafting the manuscript, performed the Rotorgene- qRT-PCR analyses and performed the IPS staining experiments with AT. JK participated in drafting the manuscript and performed the cell culture irradiations. VH and SS were responsible supervisors of the project, participated in the design of the studies, data analyses and in drafting the manuscript. All authors read and approved the final manuscript.
